# Transcriptome map and genome annotation of flax line 3896

**DOI:** 10.3389/fpls.2025.1520832

**Published:** 2025-05-16

**Authors:** Daiana A. Zhernova, Alexander A. Arkhipov, Tatiana A. Rozhmina, Alexander A. Zhuchenko, Nadezhda L. Bolsheva, Elizaveta A. Sigova, Ekaterina M. Dvorianinova, Elena V. Borkhert, Elena N. Pushkova, Nataliya V. Melnikova, Alexey A. Dmitriev

**Affiliations:** ^1^ Engelhardt Institute of Molecular Biology, Russian Academy of Sciences, Moscow, Russia; ^2^ Federal Research Center for Bast Fiber Crops, Torzhok, Russia; ^3^ All-Russian Horticultural Institute for Breeding, Agrotechnology and Nursery, Moscow, Russia; ^4^ Moscow Institute of Physics and Technology, Moscow, Russia

**Keywords:** flax, *Linum usitatissimum*, line 3896, linseed, transcriptome map, reference genome, genome annotation

## 1 Introduction

Flax (*Linum usitatissimum* L.) is valued and cultivated worldwide for its dual-purpose use as both a seed and fiber crop. It is one of the oldest domesticated and most widely used crops (Kvavadze et al., 2009). Flax genome has a diploid chromosome set 2x=2n=30 and a size ~450 Mb (Dvorianinova et al., 2022; You et al., 2023). Flax seeds are rich in omega-3 fatty acids, digestible proteins, dietary fiber, and lignans (Goyal et al., 2014). Consumption of linseed oil has beneficial effects on human health, reducing the risks of many disorders and promoting immunity (Mali et al., 2019; Saini et al., 2021; Al-Madhagy et al., 2023). In addition, flax seed oil is used for technical purposes and also serves as a highly nutritious feed for livestock (Xu et al., 2022; Yadav et al., 2024). Flax fiber goes into the production of eco-friendly textiles with high absorption capacity and composite materials (Asyraf et al., 2022; More, 2022).

Different flax varieties are grown for different purposes and vary considerably in their characteristics. Today, traditional breeding is being aided by biotechnology and molecular genetics to select individuals with the desired traits more quickly and efficiently due to high-quality genome assemblies and their annotations. Since it is the study of transcriptomes in various tissues and organs that allows us to establish the associations between a valuable characteristic and its causative region in the genome (Dmitriev et al., 2020; Guo et al., 2020).

To date, there is a great deal of disparate data on gene expression in different flax organs and tissues, under different growth conditions and at different stages of ontogeny. Much work was devoted to the study of the involvement of specific genes in important agronomic traits, resistance to biotic and abiotic stressors, the regulation of organogenesis in flax plants and other characteristics.

Agriculturally valuable traits include those related to yield and to the quality of oil and fiber. The yield and the quality of oil were the subject of great interest of many studies (Xie et al., 2019; Miart et al., 2021; Gao et al., 2022; Jiang et al., 2022; Wang et al., 2022b; Dvorianinova et al., 2023b; Pushkova et al., 2024). Since the valuable product obtained from flax is fiber, the characteristics of phloem fibers were actively studied (Roach and Deyholos, 2007; Zhang and Deyholos, 2016; Gorshkov et al., 2017; Gorshkova et al., 2018; Gorshkov et al., 2019; Galinousky et al., 2020; Mokshina et al., 2020; Petrova et al., 2021; Guo et al., 2022; Mokshina et al., 2022; Yu et al., 2022; Bao et al., 2023; Gorshkova et al., 2023; Liu et al., 2023; Ibragimova and Mokshina, 2024). In addition, transcriptome analysis largely allowed the identification of genes associated with flax plant height (Guo et al., 2021), the length of the growing season, the time of flowering, and the duration of ripening (Gao et al., 2022; House et al., 2022).

Data on flax gene expression and co-expression under suboptimal environmental conditions allowed researches to assess the association of genes with resistance to pathogen infection (Galindo-González and Deyholos, 2016; Dmitriev et al., 2017; Wu et al., 2019b; Boba et al., 2021; He et al., 2022) and abiotic stressors (Yu et al., 2014; Dmitriev et al., 2016; Dash et al., 2017; Wu et al., 2018; Krasnov et al., 2019; Wu et al., 2019a; Huang et al., 2021; Wang et al., 2021; Soto-Cerda et al., 2022; Wang et al., 2022a; Danaeipour et al., 2023; Kostyn et al., 2023; Qiu et al., 2023; Wang et al., 2023; Zhang et al., 2024).

The regulation of organogenesis in flax plays an important role in understanding the development of valuable flax traits (Saha et al., 2021; Yuan et al., 2021; Qi et al., 2023; Zhao et al., 2023). The comparative study of expression profiles of linseed and fiber flax varieties identified genes associated with flax plant type, flax oil odor, and paleohistorical data (Sveinsson et al., 2014; Povkhova et al., 2021; Yang et al., 2022).

Several synthesis articles with annotations for genome assemblies of flax varieties were published: linseed CDC Bethune (Wang et al., 2012) and fiber flax YY5 (Sa et al., 2021). However, the CDC Bethune genome contains some errors because it was assembled only from Illumina reads, which did not allow researchers to resolve its complexity (Sa et al., 2021; Dvorianinova et al., 2023a). The YY5 genome was annotated with transcriptome data of a different variety for only five samples of mature flax plants: leaf, stem, root, flower, and fruit.

In the NCBI database, the reference genome of *L. usitatissimum* is currently represented by a high-quality assembly of line 3896 (https://www.ncbi.nlm.nih.gov/datasets/genome/GCA_030674075.2/, accessed on 12 October 2024) obtained by us earlier (Dvorianinova et al., 2023a). Line 3896 belongs to the group of linseed flax and is characterized by resistance to Fusarium wilt (Rozhmina and Loshakova, 2016; Dmitriev et al., 2017) and edaphic stressor (low acidity) (Rozhmina et al., 2020), high seed yield and oil content (our observations). In the present study, we complement previous studies of line 3896 with a transcriptome map and genome annotation, which were necessary to make further progress in the field of flax genome research. Our annotation was obtained with the use of RNA-Seq data, whose positive effect on the annotation result was previously shown (Salzberg, 2019; Gabriel et al., 2024). The study of flax genome organization and gene expression will allow the development of methods to obtain improved varieties with desired traits with high efficiency. The results of the study are of use for the identification of genes and polymorphisms responsible for valuable traits and development of modern breeding technologies: genome editing, marker-assisted and genomic selection.

## 2 Materials and methods

### 2.1 Plant material

Seeds of linseed line 3896 were provided by the Institute for Flax (Torzhok, Russia). To obtain transcriptome data, we collected a set of organs (**Table 1**; **Figure 1**) of line 3896 plants at different stages of vegetation under optimal growth conditions described in the next subsection.

**Table 1:** Examined samples of flax line 3896 organs/tissues at different stages of ontogenesis.

#	ID	Title	Description
1	Lin_SAM_1 Lin_SAM_2	SAM	Shoot apical meristems (SAM) from the upper part of the shoot at 30 days after germination (DAG). Their diameter was about 0.5 mm. Pool from 10 different plants.
2	Lin_FAM_1 Lin_FAM_2	FAM	Floral apical meristems (FAM) from the upper part of the shoot at 30 DAG if the shoot reached the early bud stage. Pool from 5 different plants.
3	Lin_leaf_blade_top_1 Lin_leaf_blade_top_2	Leaf laminae of young leaves	Leaf blades of young leaves from the top of the shoot at 30 DAG. Pool from 5 different plants.
4	Lin_leaf_blade_top3_1 Lin_leaf_blade_top3_2	Leaf laminae of intermediate leaves	Intermediate leaf blades from the shoot at 3 cm from the top at 30 DAG. Pool from 5 different plants.
5	Lin_leaf_blade_top10_1 Lin_leaf_blade_top10_2	Leaf laminae of mature leaves	Mature leaf blades from the shoot at 10 cm from the top at 30 DAG. Pool from 5 different plants.
6	Lin_shoot_1-3_1 Lin_shoot_1-3_2	Stem fragments 1-3 cm from the top	Stem fragments 1-3 cm from the top at 30 DAG. Pool from 3 different plants.
7	Lin_shoot_9-10_1 Lin_shoot_9-10_2	Stem fragments 9-10 cm from the top	Stem fragments 9-10 cm from the top at 30 DAG. Pool from 2 different plants.
8	Lin_s_SAM_1 Lin_s_SAM_2	SAM of seedlings	SAM of seedlings from the upper part of the shoot between cotyledons at 5 DAG on Petri dishes. Pool from 6 different plants.
9	Lin_s_cotyledon_1 Lin_s_cotyledon_2	Cotyledons of seedling	Cotyledons of seedlings at 5 DAG on Petri dishes. There were no true leaves yet, just the cotyledons. Pool from 6 different plants.
10	Lin_s_hyp_1 Lin_s_hyp_2	Hypocotyls of seedlings	Hypocotyls of seedlings collected as the stem between cotyledons and roots at 5 DAG on Petri dishes. Pool from 6 different plants.
11	Lin_s_root_1 Lin_s_root_2	Roots of seedlings	Roots of seedlings at 5 DAG on Petri dishes. Pool from 6 different plants.
12	Lin_anther_1 Lin_anther_2	Anthers of mature flowers	Mature flower anthers (before opening) at 56 DAG. Pool from 6 different plants.
13	Lin_pistil_1 Lin_pistil_2	Carpels of mature flowers	Mature flower carpels (before pollination) at 56 DAG. Pool from 6 different plants.
14	Lin_filament_1 Lin_filament_2	Stamen filaments of mature flowers	Mature flower stamen filaments (before opening) at 56 DAG. Pool from 6 different plants.
15	Lin_petal_1 Lin_petal_2	Petals of mature flowers	Mature flower petals (before opening) at 56 DAG. Pool from 6 different plants.
16	Lin_sepal_1 Lin_sepal_2	Sepals of mature flowers	Mature flower sepals (before opening) at 56 DAG. Pool from 6 different plants.
17	Lin_flower_1 Lin_flower_2	Flowers without pedicels	Mature flowers without pedicels at 56 DAG. Pool from 4 different plants.
18	Lin_pedicel_1 Lin_pedicel_2	Pedicels of mature flowers	Pedicels of mature flowers at 56 DAG. Pool from 6 different plants.
19	Lin_capsule_3_1 Lin_capsule_3_2	Capsule at 3 DAF	Capsule without seeds at 3 days after flowering (DAF).
20	Lin_capsule_7_1 Lin_capsule_7_2	Capsule at 7 DAF	Capsule without seeds at 7 DAF.
21	Lin_capsule_14_1 Lin_capsule_14_2	Capsule at 14 DAF	Capsule without seeds at 14 DAF.
22	Lin_capsule_21_1 Lin_capsule_21_2	Capsule at 21 DAF	Capsule without seeds at 21 DAF.
23	Lin_capsule_28_1 Lin_capsule_28_2	Capsule at 28 DAF	Capsule without seeds at 28 DAF.
24	Lin_seed_3_1 Lin_seed_3_2	Seeds at 3 DAF	Seeds without capsule at 3 DAF.
25	Lin_seed_7_1 Lin_seed_7_2	Seeds at 7 DAF	Seeds without capsule at 7 DAF.
26	Lin_seed_14_1 Lin_seed_14_2	Seeds at 14 DAF	Seeds without capsule at 14 DAF.
27	Lin_seed_21_1 Lin_seed_21_2	Seeds at 21 DAF	Seeds without capsule at 21 DAF.
28	Lin_seed_28_1 Lin_seed_28_2	Seeds at 28 DAF	Seeds without capsule at 28 DAF.

**Figure 1:**
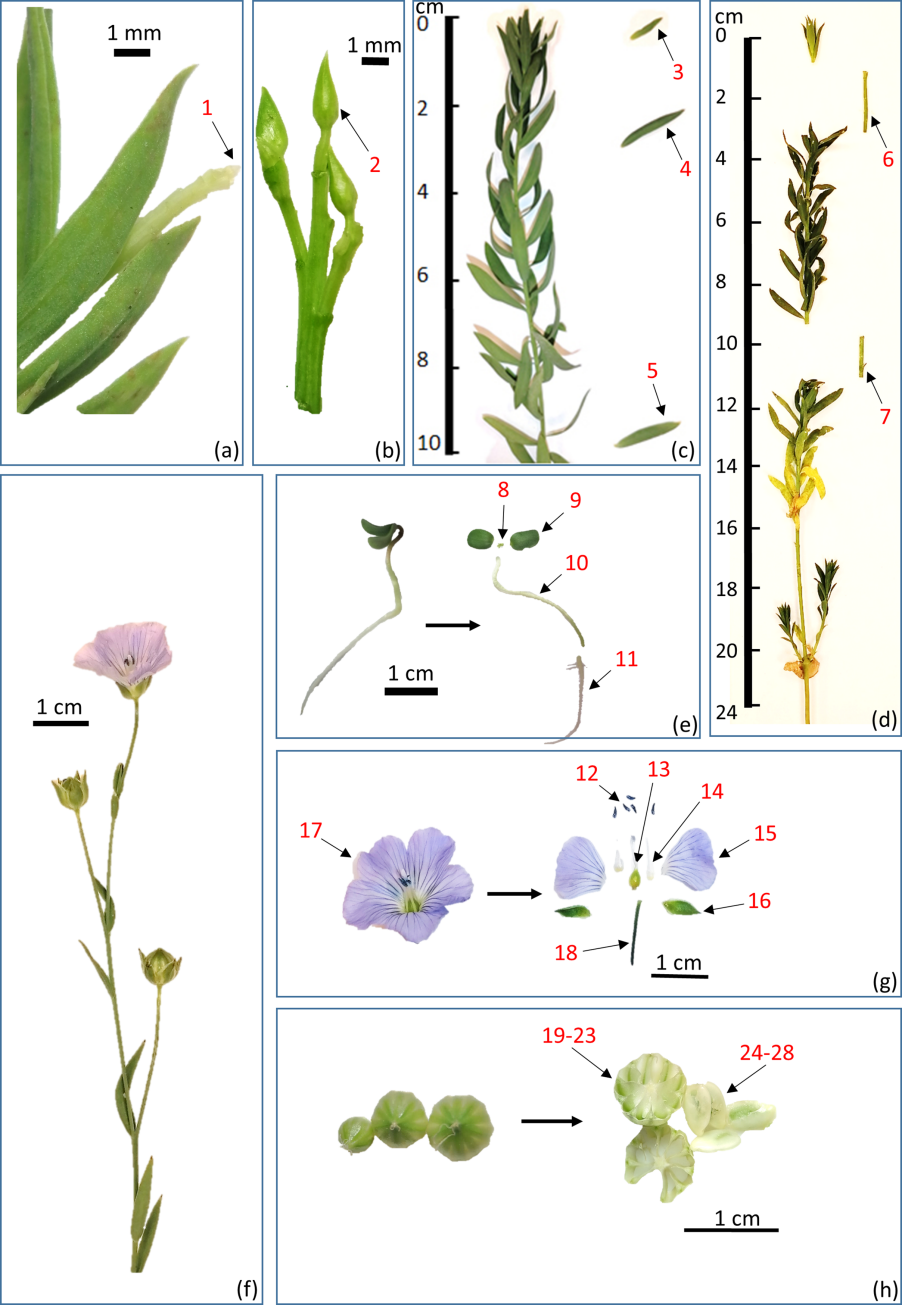
Flax organs/tissues studied by transcriptome analysis: **(a-d)** Apical parts of stem; **(e)** Seedling; **(f)** Mature plant with a flower and capsules; **(g)** Whole flower and its parts; **(h)** Capsules and seeds. 1 – SAM (shoot apical meristem); 2 – FAM (floral apical meristem); 3 – Leaf lamina of young leaf; 4 – Leaf lamina of intermediate leaf; 5 – Leaf lamina of mature leaf; 6 – Stem fragment 1-3 cm from the top; 7 – Stem fragment 9-10 cm from the top; 8 – SAM of seedling; 9 – Cotyledons of seedling; 10 – Hypocotyl of seedling; 11 – Root of seedling; 12 – Anthers of mature flower; 13 – Carpel of mature flower; 14 – Stamen filaments of mature flower; 15 – Petals of mature flower; 16 – Sepals of mature flower; 17 – Flower without pedicel; 18 – Pedicel; 19-23 – Capsule; 24-28 – Seeds. Numbers in **Figure 1** corresponds to those in **Table 1**.

### 2.2 Flax cultivation

Flax seeds were sterilized in 1% sodium hypochlorite and 96% ethanol, after which the seeds were washed twice with water and planted in 0.05% fungicide Maxim (Syngenta, Gaillon, France) solution in Petri dishes for 7 days. Seedlings were planted in the soil and continued to grow under greenhouse conditions at 20°C and ~50-70% relative humidity with regular watering.

For transcriptome analysis, a set of different organs/tissues at different development stages was collected. The plant parts and ontogenetic stages used in the study are listed in **Table 1**. Each of the samples was collected in two biological replicates. Moreover, each sample was a pool of organs from 2-10 different plants (except capsules and seeds), which is necessary to level out differences between samples and be able to capture trends common to the species (Takele Assefa et al., 2020). The flowers were marked with the date of the day it opened (day of flowering). Seeds from the same capsules were pooled. Capsules were not pooled. The age of the plants at the time of each collection and the collection conditions are shown in **Table 1**. Samples were collected in the middle of the day between 12 and 15 h under similar conditions to smooth the influence of circadian rhythms on gene expression profiles. Samples were collected in liquid nitrogen and stored at -70°C.

### 2.3 RNA isolation

Samples #1-6 and #8-18 were grinded using a TissueLyser II homogenizer (Qiagen, Hilden, Germany) with the addition of 3 ceramic beads for two minutes. The harder samples (#7 and #19-28) were homogenized using a disposable pestle inserted in a DeWALT DCD701D2 cordless drill/driver (DeWALT, Towson, MD, USA) at 1200-1500 rpm in 1.5 ml tubes in liquid nitrogen to a fine powder, without allowing the sample to thaw. RNA isolation from samples #1-18 was performed using the Quick-RNA Miniprep Kit (Zymo Research, Irvine, CA, USA). RNA isolation from capsule and seed samples (#19-28) was performed by CTAB with modifications described previously (Pushkova et al., 2024). After that, total RNA was additionally cleaned using the CleanRNA Standard kit (Evrogen, Moscow, Russia) according to the manufacturer’s protocol with a DNAase I treatment step from the RNase-Free DNase Set (Qiagen). The quality of isolated RNA was checked by horizontal electrophoresis in 2% agarose gel and using a 2100 Bioanalyzer (Agilent Technologies, Santa Clara, CA, USA), and concentrations were measured using a Qubit 4 fluorometer (Thermo Fisher Scientific, Waltham, MA, USA).

### 2.4 cDNA library preparation and sequencing on the Illumina platform

The cDNA libraries were prepared using the QIAseq Stranded mRNA Select Kit (Qiagen) according to the manufacturer’s protocol. The quality of the obtained cDNA libraries (agreement of the length of the obtained libraries with the expected one and the absence of adapter dimers) was assessed on a Qsep1-Plus capillary electrophoresis system (BiOptic, New Taipei City, Taiwan), and the concentration was evaluated on a Qubit 4 fluorometer (Thermo Fisher Scientific). The cDNA libraries (all samples from **Table 1**) were mixed equimolarly and sequenced on a NextSeq 2000 instrument (Illumina, San Diego, CA, USA) using the NextSeq 2000 P3 Reagents (100 Cycles) kit (Illumina) in 51 + 51 nucleotide format.

### 2.5 Genome assembly annotation

RNA-Seq reads were trimmed with fastp 0.23.4 using default parameters (Chen et al., 2018). Structural annotation for the flax line 3896 genome assembly (https://www.ncbi.nlm.nih.gov/datasets/genome/GCA_030674075.2/, accessed on 12 October 2024) (Dvorianinova et al., 2023a) was performed using BRAKER3 3.0.8 (Gabriel et al., 2024). This process utilized our RNA-Seq transcriptome data in combination with known Viridiplantae sequences from OrthoDB protein database for gene prediction (Kuznetsov et al., 2023). Functional annotation of the predicted genes was carried out using a local version of InterProScan 5.69-101.0 to assign functional domains and predict gene functions (Jones et al., 2014). The completeness of the annotation was assessed using BUSCO 5.7.1 in protein mode (the eudicots_odb10 dataset). Default parameters of program settings were used in the data analysis.

### 2.6 Analysis of transcriptome data

Gene expression analysis was performed with PPline (Krasnov et al., 2015) with default parameters of program settings and included the following steps:

- Alignment of the trimmed RNA-Seq reads to the annotated in the present study reference genome of flax line 3896 using STAR 2.7.2b (Dobin et al., 2013).
- Quantification of gene expression: read overlaps with annotated genomic features were counted using featureCounts 1.6.0 (Liao et al., 2014).
- Calculation of the normalized gene expression values, in counts per million (CPM), using edgeR.
- Sample normalization using TMM to account for differences in library sizes and composition between samples.

### 2.7 Gene enrichment analysis

Custom Gene Ontology (GO) annotation for line 3896 was constructed using EggNOG-mapper (–tax_scope 33090) (emapper 2.1.12, eggNOG DB version: 5.0.2) for the longest proteins in the constructed gene annotation (Huerta-Cepas et al., 2019; Cantalapiedra et al., 2021). Differential gene expression analysis was conducted for a balanced subset of flax samples: mature leaf blades from the shoot at 10 cm from the top at 30 days after germination (DAG), stem fragments 1-3 cm from the top at 30 DAG, roots of seedlings at 5 DAG, hypocotyls of seedlings at 5 DAG, mature flower carpels before pollination at 56 DAG, capsule without seeds at 14 days after flowering (DAF), seeds without capsule at 14 DAF, shoot apical meristems (SAM) of seedlings at 5 DAG. Gene expression in each tissue was compared to that in the other tissues from the subset of flax samples. Genes with FDR (QLF) < 0.05 and logFC > 1.5 were selected for gene enrichment analysis. Gene enrichment was performed using the constructed annotation, the selected gene lists, and the topGO 2.54.0 package (Alexa and Rahnenfuhrer, 2023).

## 3 Preliminary data analysis

### 3.1 RNA-seq data characteristics

This article presents data of transcriptome analysis of 28 organs/tissues of flax line 3896: 7 samples of vegetative organs of actively growing individuals, 4 samples of seedlings, 7 samples of various parts of generative organs at flowering stage, and 10 samples of fruits and seeds at different stages of maturation (**Table 1**; **Figure 1**). We set out to cover all growth stages of flax and all key events in the development of valuable flax traits. From 5.4 to 20.5 million raw reads (51 + 51 bp) were obtained for each sample on the Illumina platform (two biological replicates were sequenced for each sample). The raw data were deposited in the NCBI Sequence Read Archive (SRA) under the BioProject accession number PRJNA1172129.

After trimming, the reads were mapped to the genome assembly of line 3896, and on average ~95% of the reads for each sample were mapped (on average ~90% were uniquely mapped), confirming the high quality of the transcriptome data.

### 3.2 Genome annotation

We collected extensive data on gene expression in 28 organs/tissues of flax line 3896 at different development stages. Using the obtained transcriptome data and the Viridiplantae protein sequence database, we annotated the line 3896 genome assembly with BRAKER3: 39,055 genes and 45,154 transcripts were predicted, and 37,787 of these genes were annotated using InterProScan (**Supplementary Data Sheet S1**). The high completeness of annotation was achieved according to BUSCO (Benchmarking Universal Single-Copy Orthologs) – 95.6% (eudicots). Notably, 22.2% of all BUSCO were complete and single-copy and 73.4% were complete and duplicated. Such a high percentage of duplicated BUSCO was expected for an ancient tetraploid (Bolsheva et al., 2017).

### 3.3 Transcriptome map

Utilizing the obtained annotation of line 3896, we performed an analysis of our transcriptome data, which resulted in the identification of genes exhibiting tissue-specific and development stage-specific expression patterns within flax organs/tissues. This analysis led to the generation of a comprehensive transcriptome map for line 3896. To present the data in a convenient format for further analysis, we used PPline and RTrans (https://github.com/gskrasnov/RTrans, accessed on 17 October 2024). It was applied to evaluate the expression levels of the identified genes as read counts per million reads (CPM). Our transcriptome map is summarized in **Supplementary Table S1** and presented as a heatmap (**Supplementary Figure S1**).

### 3.4 Gene pathway enrichment analysis

During the gene pathway enrichment analysis, the following was found out. For leaves collected at a distance of 10 cm from the apex, compared to the other organs/tissues, the GO terms were represented by the processes of photosynthesis, carbohydrate metabolism, plastid organization, electron transport chain, pigment synthesis, and transmembrane transport. These processes are characteristic of the main photosynthetic organ of the plant in the active phase (Müller and Munné-Bosch, 2021; Leister, 2023). In the stem fragment, located 1-3 cm from the top, the processes of vascular tissue histogenesis, vascular and phloem transport, stem morphogenesis, and response to auxin synthesized in the apical meristem predominated. This is logical, since we were dealing with an axial organ whose main function is the transport of metabolites, and the incision was made close to the site of differentiation (Yoshida et al., 2009; Kułak et al., 2023). It was determined that the major pathways in the seedling root included the processes of water and solute transport, root hair formation and growth, response to chemical and mechanical stimuli, and metabolism of auxin, other hormones, and secondary metabolites, which are the main processes occurring in the roots of vascular plants (Vissenberg et al., 2020; Li et al., 2021; Castillo-Jiménez et al., 2023). GO analysis of the hypocotyl transcriptome revealed differential expression of genes related to pathways of amino acid biosynthesis, as well as active regulation of biosynthetic processes, brassinosteroid metabolism, and cell growth. These findings indicate the presence of active development processes in the axial organs of seedlings (Favero et al., 2021). GO analysis for flax pistil demonstrated the representation of genes that are associated with the formation and development of generative structures, pollination, and pollen tube growth. Furthermore, it demonstrated representation of genes associated with active ion metabolism, which is necessary for directed pollen tube growth (Zhou et al., 2022). GO analysis for a capsule (14 DAF) indicated that the GO terms associated with the formation of secondary cell wall, synthesis of its components, and lignification prevailed. These processes enable the preparation of dry fruits for opening and seed dispersal (Seymour et al., 2013). Additionally, catabolism of organic compounds used for cell wall construction and seed maturation is active in ripening capsules. The differential expression pattern of flax seeds (14 DAF) was dominated by processes related to seed and fruit development and maturation, lipid storage and fatty acid synthesis, as well as abscisic acid (ABA) metabolism and regulation. Oil accumulation corresponds to the primary function of the seed as a reproductive organ, and ABA regulates its maturation (Sano and Marion-Poll, 2021; Dvorianinova et al., 2023b). The shoot apical meristem displayed a hallmark pattern indicative of actively dividing cells. The process of SAM is characterized by the macromolecule biosynthesis, ribosome assembly, translation, RNA processing, DNA reparation, organelle formation, nucleosome assembly, and chromatin remodeling (Xue et al., 2020; Burian, 2021). Thus, the results of the gene enrichment analysis of the subset of flax samples in general looked logical and confirmed the adequacy of the obtained data. The results are presented in detail in **Supplementary Table S2**.

## 4 Conclusions

Flax is of great industrial and nutritional value and is therefore actively studied at the molecular-genetic level. To date, there are several flax genome assemblies, some with annotations, and many scattered gene expression data for different varieties. In this study, we used Illumina sequencing to obtain comprehensive transcriptome data for flax line 3896, whose genome is currently a reference for the species *Linum usitatissimum* L. in the NCBI database. Gene expression profiles were analyzed in 28 various flax organs/tissues at different stages of ontogenesis. With these data we were able to annotate the genome of line 3896 and generate a high-quality transcriptome map. The transcriptome map will allow the identification of genes that have a high expression level in a particular organ/tissue. Such genes may play a key role in the biological processes taking place in that organ/tissue. In addition, data on gene expression profiles during plant development can help to determine the most important time points at which the processes of interest occur. The transcriptome map also allows the determination of gene functions based not only on homology analysis, but also taking into account gene expression patterns in different organs/tissues. Thus, the transcriptome map and annotation presented in this work allow reaching a new level in the molecular-genetic studies of flax, the search for key genes responsible for the valuable traits, the development of new approaches in flax breeding and the creation of improved varieties.

## Data Availability

The datasets presented in this study can be found in online repositories. The names of the repository/repositories and accession number(s) can be found below: https://www.ncbi.nlm.nih.gov/, PRJNA1172129.

## Supplementary material

Supplementary Figure S1 Expression heatmap based on log_2_(CPM/avg.CPM) of annotated genes in 28 organs/tissues of flax line 3896.

Supplementary Table S1 Expression levels of annotated genes in 28 organs/tissues of flax line 3896 as read counts per million reads (CPM).

Supplementary Table S2 Gene enrichment analysis for a balanced subset of flax samples: mature leaf blades from the shoot at 10 cm from the top at 30 DAG, stem fragments 1-3 cm from the top at 30 DAG, roots of seedlings at 5 DAG, hypocotyls of seedlings at 5 DAG, mature flower carpels before pollination at 56 DAG, capsule without seeds at 14 DAF, seeds without capsule at 14 DAF, SAM (shoot apical meristem) of seedlings at 5 DAG.

Supplementary Data Sheet S1 Annotation of flax line 3896 genome assembly.
