# Supplementary figures and images for: Transcriptome map and genome annotation of flax line 3896
Source: Front Plant Sci. 2025 May 16;16:1520832. doi: 10.3389/fpls.2025.1520832 (PMC12122762; doi:10.3389/fpls.2025.1520832)

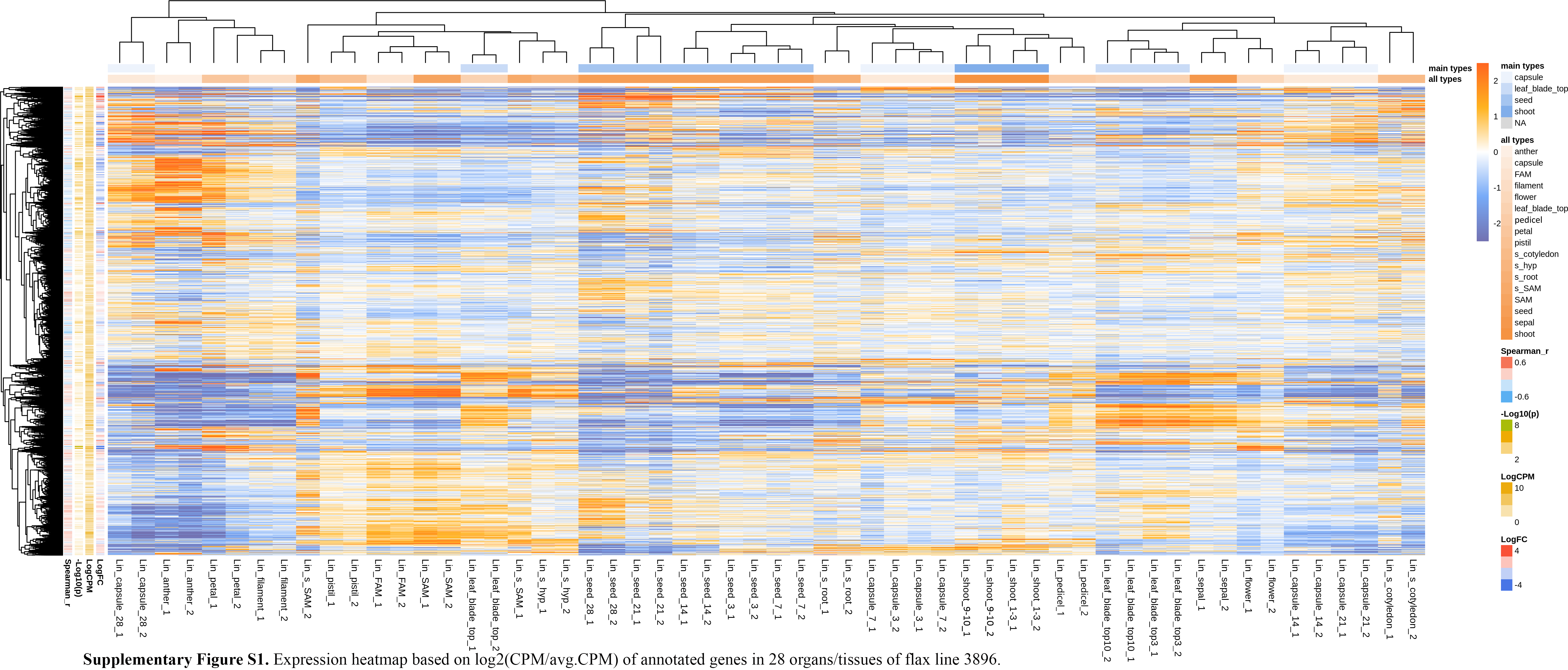

Supplement: Supplementary Figure S1 — Expression heatmap based on log2(CPM/avg.CPM) of annotated genes in 28 organs/tissues of flax line 3896. [file Image1.png]
